# Artificial Intelligence (AI) – Powered Documentation Systems in Healthcare: A Systematic Review

**DOI:** 10.1007/s10916-025-02157-4

**Published:** 2025-02-18

**Authors:** Aisling Bracken, Clodagh Reilly, Aoife Feeley, Eoin Sheehan, Khalid Merghani, Iain Feeley

**Affiliations:** 1 Royal College of Surgeons in Ireland (RCSI), 123 Stephen’s Green, Dublin 2, Ireland; 2 University College Dublin (UCD), Belfield, Dublin 4, Ireland; 3 School of Medicine, University of Limerick (UL), Castletroy, Limerick, Ireland; 4 Midlands Regional Hospital Tullamore, Arden Rd., Tullamore, Offaly Ireland

**Keywords:** Artificial Intelligence (AI), Chat GPT, Ambient intelligence, Medical documentation

## Abstract

Artificial Intelligence (AI) driven documentation systems are positioned to enhance documentation efficiency and reduce documentation burden in the healthcare setting. The administrative burden associated with clinical documentation has been identified as a major contributor to health care professional (HCP) burnout. The current systematic review aims to evaluate the efficiency, quality, and stakeholder opinion regarding the use of AI-driven documentation systems. Using the Preferred Reporting Items for Systematic Reviews and Meta-Analyses (PRISMA) guidelines a comprehensive search was conducted across PubMed, Embase and Cochrane library. Two independent reviewers applied inclusion and exclusion criteria to identify eligible studies. Details of AI technology, document type, document quality and stakeholder experience were extracted. The review included 11 studies. All included studies utilised Chat generated pretrained transformer (Chat GPT, Open AI, CA, USA) or an ambient AI technology. Both forms of AI demonstrated significant potential to improve documentation efficiency. Despite efficiency gains, the quality of AI-generated documentation varied across studies. The heterogeneity of methods utilised to assess document quality influenced interpretation of results. HCP opinion was generally positive, users highlighted ease of use and reduced task load as primary benefits. However, HCPs also expressed concerns about the reliability and validity of AI-generated documentation. Chat GPT and ambient AI show promise in enhancing the efficiency and quality of clinical documentation. While the efficiency benefits are clear, the challenges associated with accuracy and consistency need to be addressed. HCP experiences indicate a cautious optimism towards AI integration, however reliability will depend on continued refinement and validation of the technology.

## Introduction

The integration of artificial intelligence (AI) in healthcare has rapidly expanded, particularly in the domain of clinical documentation [1]. This evolution is driven by the need to improve documentation standards while also addressing health care professional (HCP) burnout. Clinical documentation burden and excessive bureaucratic tasks have been cited as leading contributors to HCP burnout [2]. AI based tools are poised to improve clinical documentation workflow and efficiency [2].

AI encompasses a variety of technologies each supporting different processes and tasks. These technologies have the potential to transform many aspects of the healthcare system [3]. Generative AI is designed to create new content based on patterns learned from text data or images. Chat generated pretrained transformer (Chat GPT) (Open AI, San Francisco, CA, USA) is a form of generative AI which utilises large language model (LLM) algorithms and advanced neural networks to generate human like text responses [4]. Ambient AI scribes, an alternative AI technology, utilise natural language processing (NLP) and machine learning (ML) algorithms to translate spoken conversation into written documentation in real time [5]. Both Chat GPT and ambient AI have shown promise in streamlining clinical documentation in a variety of settings [5–7].

The burden of clinical documentation is well-documented; evidence suggests that HCPs spend on average two hours outside the official working day on documentation tasks [8]. This administrative load not only contributes to burnout but also detracts from time that could be spent on direct patient care [9]. Moreover, the variability in the quality of documentation—ranging from omissions of critical details to inconsistent formatting—can impact patient outcomes and continuity of care [10, 11]. A reduction in documentation burden would function to improve physician burnout and overall patient care [2, 12]. These challenges have prompted the exploration of advanced technological solutions.

Despite the promising potential of AI in clinical documentation, concerns remain regarding the overall quality of AI-generated notes. Advanced AI systems have the ability to produce “hallucinations” – fictious or fabricated output which is presented as fact [13]. Hallucinations may limit the use of LLMs such as ChatGPT in clinical settings. Physicians must be aware of the limitations of generative AI models prior to their implementation in clinical settings.

This systematic review aims to evaluate the efficiency, quality, and stakeholder opinions regarding the use of AI, specifically generative and ambient AI, in clinical documentation. By providing an evidence-based analysis, this review seeks to inform healthcare providers and policymakers on the viability of adopting AI-driven documentation solutions in clinical practice.

## Methods

### Search Strategy

A search strategy to ensure retrieval of relevant articles was utilised. The search employed keywords, Medical Subject Headings (MeSH) and Boolean operators based on the intervention and setting (Table 1). The search strategy did not include specific outcomes of interest or study design as this may have limited search results. There were no boundaries placed on year of publication. Databases searched include PubMed, Embase and Cochrane Library.

**Table 1:** Search Strategy

PubMed 2,688 (08/08/24)	(“Documentation”) OR (“Medical Documentation”) OR (“Medical Notes”) OR (“Clinical Documentation”) OR (“Clinical Notes”) OR (“Charting”) OR (“Patient Note”) OR (“Patient Chart”) AND (“Artificial Intelligence AI”) OR (“Chat GPT”) OR (“Digital Scribe”) OR (“Automatic Speech Recognition”) OR (“Virtual Assistant) OR (“Ambient Intelligence”) OR (“Machine Learning”) OR (“Natural Language Processing”)
Embase 767 (08/08/24)	(“Artificial Intelligence AI”) OR (“Chat GPT”) OR (“Automatic Speech Recognition”) OR (“Virtual Assistant) OR (“Ambient Intelligence”) OR (“Machine Learning”) OR (“Natural Language Processing”) AND (“Documentation”) OR (“Medical Documentation”) OR (“Clinical Documentation”) OR (“Clinical Notes”) OR (“Charting”) [Embase]/lim NOT ([Embase]/lim AND [Medline]/lim)
Cochrane Library 241 (08/08/24)	(“Artificial Intelligence AI”) OR (“Chat GPT”) OR (“Automatic Speech Recognition”) OR (“Machine Learning”) OR (“Natural Language Processing”) AND (“Documentation”) OR (“Medical Documentation”) OR (“Medical Note”) OR (“Charting”) OR (“Patient Chart”)

### Study Eligibility and Selection Process

**Fig. 1:**
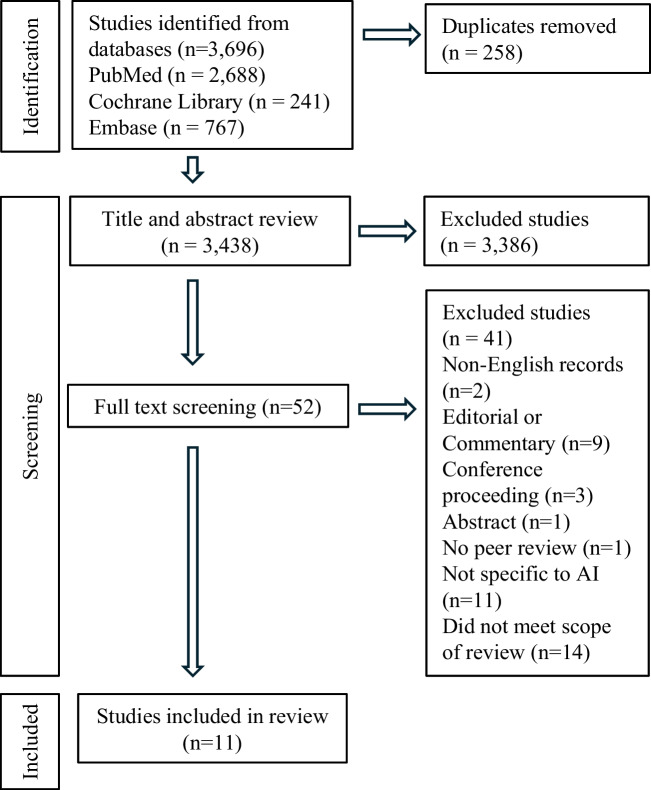
PRISMA Flowchart

Studies that met the population, intervention, comparator, outcome, study design and setting (PICOSS) criteria were included in the review (Table 2) [14]. The systematic review was conducted according to the Preferred Reporting Items for Systematic Reviews and Meta-Analyses (PRISMA) guidelines (Fig. 1) [15]. After the database search, identified references were compiled using Rayyan (Cambridge, MA, USA) a systematic review tool. Duplicate records were removed. Two independent reviewers initially screened articles for relevance based on title and abstract, studies deemed not to meet inclusion criteria were excluded. A subsequent eligibility assessment was undertaken via full text review of remaining studies.

**Table 2:** PICOSS Criteria

PICOSS	Inclusion Criteria	Exclusion Criteria
Population	Health Care Professionals (HCP) – Not limited to—Doctors, Nurses, Physiotherapists, Occupational Therapists, Pharmacists, Dentists	Administrative staff
Intervention	Use of any artificial intelligence (AI) technology for the generation of all types of clinical documentation such as progress notes, discharge summaries, handover documents, clinic letters, operation notes	Utilising AI technology for data extraction from clinical documentation Clinical documentation generation using non-AI technologies such as smart phone applications Non – clinical documentation generation utilising AI technologies
Comparison	None	None
Outcomes	Studies included must include at least one outcome measure of interest – Quality of documentation produced. Efficiency of documentation generation. Usability of AI technologies for documentation production. Stakeholder opinion of AI technology use	Outcomes focused on the evaluation of the AI technology itself rather than the evaluation of documentation produced
Study Design	All types of study design which involve primary data collection and analysis including quantitative, qualitative and mixed methods	Studies based on secondary data such as narrative reviews and systematic reviews. Non-peer reviewed articles. Conference proceedings, editorials or letters to the editor Non English Studies
Setting	Health care setting including hospital setting (wards, clinic, emergency department (ED), operating theatre) and primary care setting	Non-healthcare setting

### Data Extraction and Quality Assessment

Studies included for review were compiled in Microsoft Excel (Version 16.84, 2024) and the following data was extracted: year of publication; study design; population cohort; type of AI technology utilised; setting of use; type of documentation produced; overall conclusion of the study; details of quality of documentation produced; quality assessment tools; documentation timing; stakeholder opinion of use and details regarding hallucinations or false information. The mixed methods appraisal tool (MMAT)[16] was used to assess the quality of eligible studies.

## Results

### Characteristics of Included Studies

A total of 3,969 studies were identified following a search of included databases, following initial and full text screening 11 studies were deemed eligible for inclusion (Fig. 1). The majority of the 3,386 studies excluded during title and abstract screening did not include AI technologies in their methods or utilised AI technology for data extraction from clinical documentation rather than documentation generation (Table 3).

**Table 3:** Study Overview

Author	AI Technology	Document Type
1. Clough et al. [17]	Chat GPT	Discharge Summaries
2. Tung et al. [18]	Chat GPT	
3. Dubinski et al. [19]	Chat GPT	
- Dubinski et al. [19]	Chat GPT	Operation Notes
4. Robinson et al. [20]	Chat GPT	
5. Balloch et al. [21]	Ambient AI (*Tortus)*	Outpatient Letters
6. Dos Santos et al. [22]	Chat GPT	Care Plan
7. Baker et al. [23] *Patient History*	Chat GPT	General Documentation
8. Barrak-Corren et al. [24] *Clinical notes, handover and family letter*	Chat GPT	
9. Kernberg et al. [25] *Clinical notes*	Chat GPT	
10. Galloway et al. [26] *Not specified*	Ambient AI *(Brand Not Specified*)	
11. Owens et al. [27] *Not specified*	Ambient AI *(Dax Nuance)*	

### Documentation Quality

Documentation quality was assessed in nine of the included studies. A variety of quantitative and qualitative assessments were utilised to determine overall quality of documentation produced using ambient AI and Chat GPT technologies (Table 4).

**Table 4:** Documentation Quality

Author	Assessment Tool	Comparator	Overall Quality	"Hallucinations"
Baker et al. [23]	PDQI-9 “Good” Score ~ 36.6 “Bad” Score ~ 26.2 [28]	Chat GPT vs Typing vs Dictation	PDQI – 9 Chat GPT 35.9; Dictation 31.6; Typing 30.4 ChatGPT generated longer and more detailed documentation. Notes generated by Chat GPT had significantly higher PDQI-9 scores	36% (4/11) of Chat GPT generated notes contained fictitious elements
Kernberg et al. [25]	PDQI-9 “Good” Score ~ 36.6 “Bad” Score ~ 26.2 [28]	Chat GPT Replicates	PDQI-9 Chat GPT mean replicate score 29.7 ChatGPT-4 can consistently generate a SOAP-style note, however errors were not uncommon. Issues with uniformity and accuracy noted	Mean 23.6 errors per clinical case, with errors of omission (86%) being the most common, followed by addition errors (10.5%) and inclusion of incorrect facts (3.2%)
Balloch et al. [21]	SAIL < 18—Very poor; 19–21 – Poor; 22–24 – Fair; 25–27 – Good; 28–30 – Very good [21]	Ambient AI vs EHR	SAIL 100% of chart notes utilising AI scored > 25 43% of chart notes using EHR scored > 25 70% of letters utilising AI scored > 25 29% of letters using EHR scored > 25 SAIL assessment identified a greater than two fold increase in document quality generated using AI compared with traditional EHR use	No identified hallucinations
Clough et al. [17]	Minimum dataset	Chat GPT vs Typed	No significant difference in quality of discharge summary generated by Chat GPT and junior doctors. Both groups displayed 97% adherence to the minimum dataset	No screening for hallucinations
Barak-Corren et al. [24]	Likert Scale	Chat GPT, No quality comparison	ChatGPT clinical documents were 7.6/10 for completeness, 8.6/10 for accuracy, 8.2/10 for efficiency, and 8.7/10 for readability as assessed via a Likert scale	No identified hallucinations
Dubinski et al. [19]	Expert Opinion	Chat GPT, No quality comparison	Chat GPT can produce factually correct discharge summaries and to a lesser degree operation notes. No comparison to traditionally generated documentation	Factually incorrect data identified in one specific neurosurgical case (craniotomy)
Tung et al. [18]	Likert Scale	Chat GPT vs Typed	Letters generated by Chat GPT scored higher than written letters in terms of information provision. Letter clarity, collegiality and follow-up was to an equivalent standard to junior doctors	No identified hallucinations
Robinson et al. [20]	GIRFT Guideline	Chat GPT, No quality comparison	Chat GPT documented 78.8% of the operation details as recommended by GIRFT guidelines	No screening for hallucinations
Dos Santos et al. [22]	Nursing Care Plan Gold Standard	Chat GPT, No quality comparison	Chat GPT produced nursing care plans similar in quality to the gold standard	No screening for hallucinations

*Physician Documentation Quality Instrument (PDQI-9); Sheffield Assessment Instrument for Letters (SAIL); Getting It Right First Time (GIRFT)*

### Efficiency

For the purposes of this systematic review, "efficiency" refers specifically to the time-savings achieved through the use of various documentation systems. Efficiency of documentation practices was assessed in five of 11 studies (Table 5). Use of AI technologies resulted in more efficient documentation in all five studies. The implementation of AI technologies in both hospital and primary care settings, led to significant improvement of mean documentation time [24, 27]. The most notable gain in efficiency can be seen in the documentation of complex cases [24].

**Table 5:** Documentation Efficiency

Author	Documentation Type	Time			
Baker et al. [23]	Patient history	**Typed**	**Dictation**	**Chat GPT**	
		96.8 s	43.7 s	69.8 s	
		No statistically significant difference was found in efficiency score between ChatGPT and dictation			
Balloch et al. [21]	Outpatient Appointment—Progress Note and Letter		**EHR**		**Ambient AI**
		**Time in conversation**	9 min 21 secs		8 min 53 secs
		**Total Time with Patient**	12 min 14 secs		9 min 01 secs
		Consultations using AI were significantly shorter overall equating to a 26.3% time-saving. Mean total time spent in conversation was not significantly different			
Barak-Corren et al. [24]	Inpatient Progress Note		**EHR**		**Chat GPT**
		**Case 1**	3 min 14 secs		3 min 10 secs
		**Case 2**	2 min 45 secs		1 min 34 secs
		**Case 3**	3 min 46 s		3 min 33 s
		**Case 4**	6 min 00 secs		3 min 50 s
		The greatest time saving was identified for the most complex clinical case with a mean time saving of 2 min and 10 s or 36%			
Dubinski et al. [19]	Discharge Summary and Operation Note		**Dictation**		**Chat GPT**
		**Discharge Summaries**	15 – 21 min		2.3 – 4.6 min
		**Operation Notes**	13.1 – 21 min		2.7 – 5.1 min
Owens et al. [27]	Primary Care Patient Encounters		**EHR**		**Ambient AI**
		**Patient – Doctor encounter**	5.9 min		1. 8 min
		After Ambient implementation average documentation time per encounter was significantly reduced by 28.8%			

### Stakeholder Experience

AI technology usability was assessed in four of the 11 studies (Table 6). The impact of AI technology use of HCP workflow was assessed utilising a variety of tools. AI technology has been shown to improve documentation experience in both inpatient [24] and outpatient settings [21, 27]. Overall, HCPs reported an improved ease in the documentation process with the use of Chat GPT and ambient intelligence in all four studies [21, 24, 26, 27].

**Table 6:** Impact on HCP Workflow

Author	Type of AI	Assessment Tool	Impact on HCP Workflow
Balloch et al. [21]	Ambient AI	NASA – TLX	Clinicians reported their clinic experience was less physically and mentally demanding using AI Clinicians felt less rushed There was an overall perception that clinicians had to input less effort to achieve a desirable performance
Barak-Corren et al. [24]	Chat GPT	Likert Scale	When using Chat GPT clinicians reported less effort was required for complex tasks
Galloway et al. [26]	Ambient AI	Likert Scale	A significant proportion of clinicians reported an improvement in the ease of the documentation process with the use of the AI tool
Owens et al. [27]	Ambient AI	OLBI	Increased use of Ambient AI was associated with improved burnout scores. High adoption rate of AI had significantly lower scores on the OLBI-D score which drove a trend to reduced total score (OLBI-T)

*NASA Task Load Index (NASA- TLX); Oldenburg Burnout Inventory (OLBI); Disengagement (OLBI-D); Total (OLBI-T)*

User opinion on AI technology use was assessed in three studies, two of these studies employed the use of ambient intelligence. User opinion was assessed using both qualitative [24] and quantitative assessment (Table 7) [21, 26].

**Table 7:** Stakeholder Opinion

Author	Type of AI	Documentation	Stakeholder Opinion	
Balloch et al. [21]	Ambient AI	Outpatient Appointment—Progress Note and Letter	Positive experiences linked to ease of use, a simple user interface, time efficiency, structured formatting of the clinic note, and the aid to note taking	Concerns focused on the accuracy of information generated by the AI tool. Loss of narrative in letters or inappropriate tone
Barak-Corren et al. [24]	Chat GPT	Inpatient Progress Note	Physicians reported improved efficiency and less effort in documenting process	Concerns related to inaccuracies and quality as a consequence
Galloway et al. [26]	Ambient AI	General Clinical Documentation	Reported improved satisfaction with documentation process, overall well-being and an improved patient experience	No concerns noted

### Quality Assessment

The included 11 studies were assessed using the mixed method appraisal tool (Table 8). Studies using either quantitative or qualitative methods were assessed out of five criteria. The three studies utilising mixed methods were assessed on 15 criteria (qualitative, quantitative and mixed criteria). The overall quality score of mixed method studies could not exceed the individual lowest component [29]. Five of the 11 included studies meet 100% (5/5) of assessed criteria. Four of the 11 studies meet only 60% (3/5) of assessed criteria. No study scored lower than 60% (3/5).

**Table 8:** MMAT

Author	Method	MMAT
Clough et al	Mixed	****
Tung et al	Quantitative	*****
Dubinski et al	Quantitative	***
Robinson et al	Quantitative	***
Balloch et al	Mixed	*****
Dos Santos et al	Qualitative	*****
Baker et al	Quantitative	*****
Barrak-Corren et al	Mixed	***
Kernberg et al	Quantitative	****
Galloway et al	Quantitative	****
Owens et al	Quantitative	***

## Discussion

AI driven clinical documentation presents a promising avenue for enhancing efficiency and reducing the administrative burden on HCPs. This systematic review synthesised findings from 11 studies examining the use of AI, particularly Chat GPT and ambient AI for various forms of clinical documents. The results highlight both the potential benefits and challenges associated with implementing AI-driven documentation systems. While AI has demonstrated the ability to improve documentation efficiency and, in some cases, quality, concerns remain about the reliability and accuracy of these technologies.

The included studies reflect a growing interest in the application AI-driven documentation systems. Notably, nine of 11 studies were published in 2024, highlighting the novelty and rapid evolution of this field. The predominance of Chat GPT and ambient AI technologies in these studies suggests a focus on generative and real-time documentation tools. The diverse range of documentation types generated highlights the versatility of AI documentation systems.

MMAT assessment revealed high methodological quality in seven of 11 included studies (80–100% of criteria met). Four studies met 60% of criteria, primarily due to concerns regarding the appropriateness of the chosen sample population or sampling strategy. To enhance the clinical applicability of future research, studies should prioritise the inclusion of HCPs whose roles directly involve documentation and include a broader range of clinical scenarios.

## Documentation Quality

The quality of AI-generated documentation remains a critical area of evaluation. The included studies reported mixed outcomes. Six of nine studies report AI-generated documents meeting or surpassing traditional documentation standards, however three of these studies did not screen for the inclusion of fictitious information or 'hallucinations’. The presence of "hallucinations" or fictitious information in three studies utilising Chat GPT raises concerns regarding safe implementation in clinical settings. Hallucination rates have been documented to vary between 3% – 28% in current literature [30, 31]. The output generated by Chat GPT is dependent on the complexity and clarity of the input, continuous evaluation of generated output will be required to ensure safe implementation in clinical settings [32, 33].

The variation in quality assessment tools utilised in included studies limits direct study comparison. Inter-user variability has been reported with the use of the physician documentation quality instrument (PDQI-9 tool) [34] When compared to previous literature AI-driven documentation varied between “bad (29.7)” and “excellent (35.6)” highlighting the variability in documentation produced by AI systems [23, 25, 28]. The disparity in documentation quality was also identified across various document types. Sheffield Assessment Instrument for Letters (SAIL) assessment of outpatient letters generated by ambient dictation identified a greater than two fold increase in document quality compared to standard EHR use [21]. However, operation notes generated by Chat GPT were determined by senior clinicians to contain fictious information and of variable quality [19]. This suggests that while AI can enhance certain aspects of documentation, its reliability is not yet consistent across all clinical scenarios. Refinement of these technologies will be required before widespread implementation.

## Efficiency

AI technologies have demonstrated substantial potential to improve the efficiency of clinical documentation. Across all studies, the use of AI technology reduced the time required for documentation. The most notable gains in efficiency were found with the use of ambient intelligence and the documentation of complex clinical cases. This time-saving aspect of AI is crucial as it directly impacts clinician workload and time available for patient care. Baker et al. identified dictation as a non-significant faster alternative to Chat GPT; authors concluded that the combination of dictation and chat GPT would create the greatest efficiency gains [23]. This conclusion reached by Baker et al. is evident in ambient AI. This technology positions itself as a hybrid between generative AI and dictation to consistently deliver documentation in an efficient manner [21, 27].

Burnout among HCPs, characterised by emotional exhaustion and depersonalisation has been closely linked to documentation burden and extensive EHR use [2, 35]. Given that documentation demands are a key contributor to burnout, the consistent efficiency gains provided by AI-driven documentation systems offers a promising solution to reduce the incidence of burnout among HCPs. However, any efficiency gains must be viewed in light of the quality of documentation produced.

## Stakeholder Experience

Stakeholder experiences and opinions on AI-driven documentation systems are generally positive, yet nuanced. HCPs frequently reported enhanced ease of use and a reduced task load, further supporting the implementation of AI-driven documentation systems as a method to alleviate documentation burden and HCP burnout. However, despite these benefits, concerns persist regarding the accuracy and structure of AI-generated documentation. The findings of this review are mirrored in previous literature as 96% of participants in one study identified data quality concerns as a major challenge in the short-term implementation of AI in the healthcare setting [36]. While stakeholders appreciate the efficiency and structured formatting provided by AI tools, scepticism remains regarding reliability and the potential loss of narrative detail in clinical notes. The widespread implementation of AI in clinical settings will likely hinge on how effectively these issues are resolved. Many barriers have been identified to the widespread implementation of AI technologies in healthcare, however previous literature has emphasised the inevitability of its adoption [3]. This proposed inevitability necessitates the need to address accuracy and quality concerns to ensure AI-driven documentation systems are reliable and valid.

## Limitations

This systematic review has several limitations that should be considered when interpreting the findings. The technologies assessed in the included studies are relatively new, and their capabilities and limitations may change rapidly as AI technology evolves. The studies included used a wide range of tools and methods to assess outcomes. This heterogeneity in assessment methods adds complexity to the interpretation of results and may affect the consistency of the findings across studies. Several studies highlighted the issue of AI-generated "hallucinations” which presents a significant risk in clinical documentation. However, the review included studies with varying degrees of scrutiny for these errors, and some studies did not screen for hallucinations at all. This inconsistency in reporting may lead to an underestimation of the potential risks associated with AI-generated documentation.

## Conclusion

While AI technologies like Chat GPT and ambient AI show promise in enhancing the efficiency and quality of clinical documentation, significant challenges remain. The variability in documentation quality undermines efficiency gains. Continued research and development are needed to refine AI tools, improve their reliability, and ensure that they can consistently meet the high standards required in clinical documentation. As the field progresses, careful consideration of both the benefits and limitations of AI in healthcare will be crucial for its successful integration into clinical practice.

## Data Availability

No datasets were generated or analysed during the current study.

## Abbreviations

AI: Artificial Intelligence
HCP: Health Care Professional
LLM: Large Language Model
NLP: Natural Language Processing
ML: Machine Learning
MMAT: Mixed Methods Appraisal Tool
PDQI: Physician Document Quality Instrument
SAIL: Sheffield Assessment Instrument for Letters
GIRFT: Getting It Right First Time
